# The Body Across Adulthood: On the Relation Between Interoception and Body Representations

**DOI:** 10.3389/fnins.2021.586684

**Published:** 2021-02-24

**Authors:** Simona Raimo, Maddalena Boccia, Antonella Di Vita, Maria Cropano, Cecilia Guariglia, Dario Grossi, Liana Palermo

**Affiliations:** ^1^Department of Psychology, University of Campania “Luigi Vanvitelli”, Caserta, Italy; ^2^Department of Psychology, “Sapienza” University of Rome, Rome, Italy; ^3^I.R.C.C.S. Santa Lucia Foundation, Rome, Italy; ^4^Department of Medical and Surgical Sciences, Magna Graecia University of Catanzaro, Catanzaro, Italy

**Keywords:** adulthood, body schema, body structural representation, body semantics, interoception

## Abstract

Interoceptive information plays a pivotal role in building body representations (BR), but the association between interoception and the different types of BR in healthy individuals has never been systematically investigated. Thus, this study aimed to explore the association between BR and interoceptive sensibility (IS) throughout adulthood. One hundred thirty-seven healthy participants (50 aged from 18 to 40 years old; 50 aged from 41 to 60 years old; and 37 over 60 years old) were given a self-report tool for assessing IS (the Self-Awareness Questionnaire; SAQ), and a specific battery including tasks evaluating three different BR (i.e., the body schema, using the Hand Laterality Task; the body structural representation, using the Frontal Body Evocation task, FBE; and body semantics, using the Object-Body Part Association Task) as well as control tasks (i.e., tasks with non-body stimuli). The older age group (aged over 60 years old) showed lower performances on the tasks probing the body schema and body structural representation than younger groups (aged 18 to 40 and 41 to 60 years old). More interestingly, worse performances on a task assessing the body schema were significantly associated with higher IS with older age, suggesting that higher awareness of one’s inner body sensations would decrease the plasticity of this BR. These findings are interpreted according to the neuropsychological model of BR development and the effects of aging on the brain.

## Introduction

Interoception refers to the ability to perceive one’s own physical sensations related to internal organ functioning, such as heartbeat, itch, respiration, and satiety (Vaitl, 1996; Cameron, 2001; Craig, 2002; Barrett et al., 2004). It is a multidimensional process that usually occurs outside of conscious awareness, but that may be consciously experienced during instances of homeostatic perturbation (i.e., interoceptive processes, Garfinkel et al., 2015; or interoceptive awareness, Khalsa et al., 2018). Studies that have sought to identify the neural substrates of interoceptive processing point to the pivotal role of the insula, particularly of the anterior insular cortex (Craig, 2002).

A more coherent nomenclature of different components of interoceptive processing (see Khalsa et al., 2018) has been developed, reflecting the need for operationalization in neuroscience and clinical practice. It mainly distinguishes between the *interoceptive attention* (i.e., the process of observing internal bodily sensation), the *interoceptive accuracy* or *interoceptive sensitivity* (i.e., the process of correctly and precisely monitoring the sensation as assessed by comparisons between subjective and objective indices), and the *interoceptive sensibility* (IS; i.e., the self-perceived tendency to focus on interoceptive signals, representing a trait-like feature).

Many studies have shown the role of interoception in cognitive functioning (e.g., in decision-making, memory, and emotion processing) and that its dysfunction is an essential component of different mental health conditions (Khalsa et al., 2009, 2018).

### Body Representations and Interoception

The processing of interoceptive information contributes to our sense of body ownership (Berlucchi and Aglioti, 2010) that refers to the feeling that my body belongs to me and “presumably depends on afferent sensations arising within the body itself, but also on the coherence of current sensory input with pre-existing cognitive representations of the body” (Costantini and Haggard, 2007, p. 230). Body ownership is relevant for body representations (BR), since it would act as an essential source of information for the internal BR state that provides forward models generating sensory predictions during voluntary action (Kilteni and Ehrsson, 2017; Grechuta et al., 2019); likewise, BR are relevant for body ownership, since the self-identification of body parts would be achieved through a dynamic multisensory integration process of peripheral inputs interpreted in the context of high-order BR (Makin et al., 2008).

A seminal study by Tsakiris et al. (2011) has nicely shown how interoceptive processing specifically modulates body ownership. They observed a negative correlation between the ability to detect one’s own heartbeat (i.e., interoceptive accuracy) and the Rubber Hand Illusion (RHI), such that participants with lower interoceptive accuracy showed a stronger RHI measured both behaviourally and physiologically (i.e., drop in skin temperature). On the other hand, a study by Filippetti and Tsakiris (2017) investigated how changes in body ownership specifically modulates interoceptive processing. They demonstrated that after being exposed to RHI, individuals with initially lower levels of interoceptive accuracy improved the performance at a standard heartbeat-counting task, suggesting that the process of a bodily illusion, biasing the experience of ownership toward an artificial and external limb such as the rubber hand, would improve the ability to accurately detect internal bodily signals in individuals with low interoceptive accuracy. Following this finding, Suzuki et al. (2013) found that participants exposed to a virtual RHI experienced an increased illusion during synchronous cardio-visual feedback with one’s own heartbeat compared to asynchronous feedback, confirming the role of interoceptive processing in body ownership. On a similar fashion, several studies focused on the effect of pleasant affective touch, known to engage interoceptive processing (Crucianelli et al., 2013; Lloyd et al., 2013; van Stralen et al., 2014), showing that the pleasant touch (i.e., slow velocity on hairy skin) enhanced the RHI.

At odds with studies on interoception and body ownership mentioned above, less attention has been paid to the role of interoception in building different high order cognitive BR.

According to the dyadic taxonomy, high order cognitive BR can be broadly classified in *body schema*, consisting of a sensorimotor representation of the body that guides actions and enables the body to unconsciously adjust the posture and movement (i.e., an action-oriented BR); and *body image*, grouping all the other perceptual, conceptual or emotional representations about the body that are not used for action (i.e., a non-action oriented BR) (Paillard, 1999; Di Vita et al., 2016).

Subsequently, Schwoebel and Coslett (2005) proposed a triadic taxonomy, further subdividing the concept of body image into *body structural representation*, a visuo-spatial body map of the body that includes information about boundaries of the body parts and their spatial relations; and *body semantics*, a representation that includes names and functions of the body parts and their associations with objects.

While the dyadic and triadic taxonomies do not explicitly refer to the role of interoception in building BR, a study by Longo et al. (2010) is particularly relevant in this context.

Following Longo et al. (2010), Longo (2016), BR can also be distinguished into those mediating somatoperception, which refers to the higher-level percepts about the body, and those mediating somatorepresentation, which refers to the abstract knowledge and beliefs about one’s own body and bodies generally. While the body schema is somatoperceptual, the body structural representation and the body semantics are somatorepresentational. According to Longo et al. (2010), critical elements of somatoperception are also the “interoceptive percepts about the nature and state of the body itself” (p. 655). Thus, the processing of interoceptive information can be particularly relevant for building up the body schema. In any case, a recent review of behavioral and neuroimaging studies on non-clinical and clinical populations has also underlined that interoceptive processing might contribute to building up the body image (Badoud and Tsakiris, 2017).

Data from lesion studies on brain-damaged patients with BR and bodily self-consciousness disorders also support the pivotal role of the interoceptive processing in BR. For example, Heydrich and Blanke (2013) found the involvement of the insula, that is a crucial area for processing the body’s physiological condition (Craig, 2002), in patients with heautoscopy (i.e., an autoscopic phenomenon in which the person experiences seeing a second own body in the extrapersonal space), suggesting that this bodily self-consciousness disorder arises from the disintegration of the visuo-somatosensory signals with the interoceptive ones. Similarly, damage to the insula has been found in patients with BR disorders defined *disturbed sensation of limb ownership* (e.g., asomatognosia, somatoparaphrenia) (Baier and Karnath, 2008).

Thus, current evidence points toward a key role of interoceptive processing in the complex formation of all BR.

### The Effect of Age on Interoception and Body Processing

As underlined by Murphy et al. (2018), the relevance of interoception for cognitive functioning and health has stimulated the study of how it can vary across the lifespan. For example, Khalsa et al. (2009), using a heartbeat detection paradigm with participants ranging in age from 22 to 63 years, found that older adults had poorer detection of their heartbeats than younger and middle-aged adults (i.e., poorer interoceptive accuracy). More recently, Murphy et al. (2018), examining IS from young to very late adulthood (until 90 years of age) by means of a self-report measure (i.e., the short version of the Body Perception Questionnaire; Porges, 1993), found that IS declines with age and suggested that the interoceptive decline due to aging could account for some age-related cognitive impairments (Murphy et al., 2018).

Aging also seems to have an impact on BR. Indeed, the subjective component of body-ownership, investigated experimentally in the lifespan using the RHI, would seem to follow a U-shape curve since young and older adults showed a stronger subjective experience of illusion than the middle-aged (Marotta et al., 2018). This finding suggests that the flexibility of BR would change across the human lifespan.

Cholewiak and Collins (2003) found that the ability to localize touch on body parts decreased with advancing age, which is consistent with an effect of aging on the body structural representation. Similarly, studies investigating the ability to mentally rotate a specific body part (Personnier et al., 2008; Skoura et al., 2008; Saimpont et al., 2009), that is a task probing the body schema (Parsons, 1994), found lower accuracy and slower reaction times in older adults.

### Plan of the Study

So far, little attention has been paid to the association between BR and interoceptive processing across the adult lifespan.

Here, to provide a better understanding of the relation between the interoceptive processing, in terms of IS, and BR during adulthood, a sample of healthy adults in different age groups was given (i) a self-report questionnaire to evaluate IS; (ii) specific tasks tapping three different BR (i.e., body schema, body structural representation, and body semantics), and (iii) control tasks (i.e., tasks withouth bodily stimuli but superimposable to the BR tasks in terms of stimuli presentation and response modality) to disentangle the contribution of other cognitive processes (i.e., visual processing, mental imagery, visuo-spatial attention or decision making) necessary to perform the BR tasks.

**FIGURE 1:**
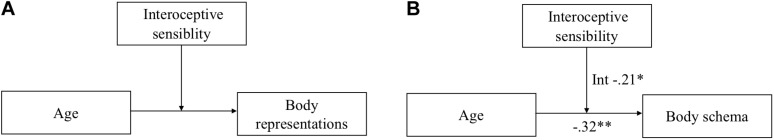
The moderation effect of the interoceptive sensibility on age’s direct effect on body representations. Hypothesized **(A)** and significant outcome **(B)** model.

Based on the previous studies reviewed above, we hypothesized that the relation between age and all BR (i.e., body schema, body structural representation, and body semantics) was affected by IS, as a moderating variable between age and BR (see Figure 1A). Indeed, interoceptive information processing declines with age, and can be relevant in building different BR.

## Materials and Methods

### Participants

One hundred thirty-seven healthy individuals participated in this study. They were grouped according to adulthood stages (Collins English and dictionary, 1994; Besedeš et al., 2012; Noh et al., 2015) in three age bands: young adulthood, aged from 18 to 40 years old (including 25 females and 25 males); middle adulthood, aged from 41 to 60 years old (including 30 females and 20 males); and older adulthood, aged over 60 years old (including 26 females and 11 males). Since the sample size was predetermined by study constraints, we performed a sensitivity power analysis that showed that a small effect size of at least Cohen’s *d* = 0.27 was required to observe a significant difference between the three age groups at an alpha level of *p* < 0.05 with 0.80 power. Thus, the study would not be able to reliably detect effects smaller than Cohen’s *d* = 0.27. Participants were recruited through personal contacts and by word of mouth from “Magna Graecia” University, Catanzaro (Italy), and from the Psychology Department of University of Campania “Vanvitelli,” Caserta (Italy). All participants were native Italians, had no current mental health disorder, such as depression and anxiety, according to the Diagnostic and Statistical Manual of Mental Health Disorders, 5th Edition (DSM-5; American Psychiatric Association, 2013), and obtained normal age- and education-adjusted scores on the Mini Mental State Examination (MMSE; Folstein et al., 1975) according to the Italian normative data (Magni et al., 1996) and on the Raven’s Colored Progressive Matrices (Raven, 1938) according to the Italian normative data (Spinnler and Tognoni, 1987), that excluded the presence of general cognitive impairment and deficit in abstract reasoning. All participants signed informed consent to participate in the study and did not receive any payments for their participation. The study was designed in accordance with the ethical standards laid down in the 1964 Declaration of Helsinki and was approved by the local ethics committees (Calabria Region Ethical Committee, Catanzaro, Italy, and Ethical Committee of the University of Campania “Vanvitelli,” Caserta, Italy). Descriptive statistics of the three age groups are reported in Table 1.

**TABLE 1:** Descriptive statistics by age groups.

	Participants aged 18 to 40 years		Participants aged 41 to 60 years		Participants aged over 60 years	
	Mean ± SD	Range	Mean ± SD	Range	Mean ± SD	Range
Age	33.98 ± 4.26	18–40	48.14 ± 4.16	41–60	69.27 ± 8.53	61–84
Sex (F/M)*	24/25		30/20		25/12	
MMSE	NA	NA	NA	NA	25.66 ± 4.60	13–30
Raven’s Coloured Progressive Matrices (age- and education- adjusted scores)*	26.24 ± 4.29	19.2–32.4	26.44 ± 4.10	19.3–31.3	26.92 ± 5.05	19.5–36.3

SD, standard deviation; NA, not assessed; MMSE, mini mental state examination.*Sex and age- and education- adjusted scores of Raven’s Colored progressive Matrices are not statistically different among groups (sex: χ^2^ = 2.78, *p* = 0.248; Raven’s Coloured progressive Matrices: *H* = 0.41 *p* = 0.812).

### Behavioural Testing

#### Assessment of IS

To evaluate IS (how frequently individuals feel signals arising from their own body), participants completed a pen-and-paper version of the Self-Awareness Questionnaire (SAQ; Longarzo et al., 2015). The questionnaire included 35 items to be rated on a five-point Likert scale (from 0 = never to 4 = always). The total score was the sum of the responses of all 35 items providing a score range of 0 to 140, with higher scores indicating higher levels of IS. The SAQ shows good internal consistency (Cronbach’s alpha ranged between 0.83 and 0.90), as found in wide samples (aged from 18 to 72 years) of Italian and English healthy adults (Longarzo et al., 2015; Hughes et al., 2019) and has been used with non-clinical as well as with clinical populations (e.g., Raimo et al., 2019a; Teghil et al., 2020).

#### Assessment of BR

According to the triadic taxonomy of Schwoebel and Coslett (2005) the assessment of BR was performed using a specific battery that included the evaluation of three distinct types of BR: the body schema, the body structural representation, and the body semantics. This battery also includes control tasks and was used in a previous study on the development of BR in healthy children and young adults (see Raimo et al., 2019b) and in a previous study on adults with brain damage (Boccia et al., 2020). Here we used for the first time the same apparatus and procedures in a sample of older adults.

##### Assessment of the body schema

The body schema was assessed using a hand mental rotation task (the Hand Laterality Task, adapted and simplified from Parsons, 1987; see Raimo et al., 2019b). In this task, participants were asked to make, as rapidly and accurately as possible, laterality judgment (“a left or a right hand?”) of a single hand (20 stimuli, 10 left, and 10 right stimuli) that could be presented in five different angles of rotation (0, 45, 90, 270, and 315 degrees). In the correspondent control task (the Object Laterality Task, see Raimo et al., 2019b), participants were asked to make, as rapidly and accurately as possible, laterality judgment (“a left or a right hand?”) of a flower with a leaf positioned at the right or at the left base of the stem (20 stimuli, 10 left, and 10 right stimuli) that could be presented in five different angles of rotation (0, 45, 90, 270, and 315 degrees). In both tasks, the stimuli presentation order was the same for all participants and the accuracy corresponded to the sum of correct responses digitally recorded; thus, individual scores ranged from 0 to 20, with higher scores indicating better performance.

##### Assessment of the body structural representation

The body structural representation was assessed using a computerized version of the “Frontal Body Evocation task” (FBE) (Daurat-Hmeljiak et al., 1978; see Raimo et al., 2019b). Although this test has been developed for use with children, it has been extensively used in literature to assess BR in samples of adults (e.g., adults with brain damage: Guariglia et al., 2002; Di Vita et al., 2017, 2019; amputee patients: Palermo et al., 2014, 2018). After watching the picture of a child for 10 s, participants had to re-locate one at time nine specific body parts (left or right leg, left or right hand, left or right arm, left or right part of the chest, and the neck), dragging it with a finger on a touchscreen where only the head of a child was depicted as a reference. Body parts were displayed one at a time on the screen. The computer recorded the position of the body part located by the participant, and then a new body part was shown (number of trials = 9). The control task involved the visuo-spatial processing of non-body related stimuli (the Christmas Tree Task, see Raimo et al., 2019b). After watching the picture of a Christmas tree for 10 s, participants had to re-locate one at time nine specific parts of the tree (left or right lower branches, left or right middle branches, left or right lower branches with trunks, left or right parts of the jar, and the top), dragging it with a finger on a touchscreen where only the star tree topper was shown as a reference. Participants were presented with one specific Christmas tree part at a time. The computer recorded the position of the Christmas tree part located by the participant, and then a new part was shown (number of trials = 9). In both tasks, accuracy was computed in terms of millimeters of deviation from the exact location of the body/tree parts from the correct locations. Thus, a smaller deviation in mm indicated better performance, whereas a higher deviation in mm indicated worse performance.

##### Assessment of body semantics

Body semantics was assessed using an Object-Body Part Association Task (adapted from Fontes et al., 2014; see Raimo et al., 2019b). In this task, participants were asked to correctly associate an object (e.g., hat) with the related part of the body, choosing between two options (e.g., head and foot). The task included 20 stimuli. The control task, that is the Object-Room Association Task, involved the semantic processing of non-body related stimuli. Participants were asked to correctly associate an object (e.g., armchair) with a room, choosing between two options (e.g., living room and bathroom). The control task included 20 stimuli. In both tasks, the accuracy that corresponded to the sum of correct responses was digitally recorded; thus, individual scores ranged from 0 to 20, with higher scores indicating better performance.

### Procedure

All participants completed the cognitive assessment and the entire battery of body and control tasks in a quiet experimental room at the Laboratory of Neuropsychology of University of Campania “Luigi Vanvitelli” and at the Laboratory of Cognitive Processes of ‘Magna Graecia’ University. First, participants were given, in this order, the MMSE (Folstein et al., 1975), the Raven’s Colored Progressive Matrices (Raven, 1938) and the SAQ (Longarzo et al., 2015). Subsequently participants performed the computerized battery of body and control tasks on a laptop (13.3” display) equipped with a touch screen monitor. For these tasks, participants were invited to sit on the chair in front of a desk with the laptop placed upon it. During testing, the participants were instructed to maintain the same position. No time limit was imposed, but they were solicited to respond immediately after the presentation of the stimulus. The order of tasks was counter-balanced across participants, but the presentation order of stimuli was consistent within all tasks.

## Statistical Analysis

The normality of data distribution was tested using the Kolmogorov-Smirnov test. Due to non-normal distribution of continuous variables (BR and control tasks and SAQ total scores), non-parametric analyses were performed.

The presence of differences on BR and IS due to the age group was evaluated using Kruskal-Wallis tests. When appropriate, pairwise comparisons were then performed by means of Mann–Whitney *U*-tests.

The next step in our data analyses involved correlation analyses to explore the association between IS and BR across the adult lifespan. First, correlations between age and SAQ total scores, and between age and the BR and control tasks were performed on the whole sample using Spearman’s rank correlations. Secondly, correlations between the SAQ total scores and the BR tasks were performed separately for the three age groups and partial correlations between the SAQ total scores and the scores on the three BR tasks controlling for age (i.e., as covariate) on the whole sample were performed. Moreover, to evaluate a possible association between IS and the BR, taking into account other cognitive functions required to perform the body tasks, partial correlations were performed between SAQ scores and the unstandardized residuals of the ranks of the body tasks on the ranks of the control tasks (i.e., the unstandardized residuals of the Hand Laterality Task scores on the Object Laterality Task scores, the unstandardized residuals of the FBE scores on the Christmas Tree Task scores; the unstandardized residuals of the Object-Body Part Association Task scores on the Object-Room Association Task scores), controlling for age (i.e., as covariate).

Finally, moderation analyses were conducted using the bootstrapping technique to assess the moderating role of IS in the relation between aging and BR. The bootstrapping moderation analysis was performed using the PROCESS macro for SPSS (Hayes, 2013), a software used for moderation, mediation, and conditional process analyses that utilizes a regression-based path analytic framework or ordinary least squares to estimate moderation models (Hayes, 2013). Thus, to examine the strength of the relation between age and BR under different values of IS, the age was inputted as the independent variable, BR task scores (Hand Laterality Task, FBE, and Object-Body Part Association Task scores) were inputted as the outcome variables, and SAQ scores were inputted as the moderator variable. Significant moderation effects were followed by models controlling for cognitive abilities (Raven’s Colored Progressive Matrix) to assess the extent to which IS’s influence was independent of this covariate.

The significance level was set at alpha level <0.05, and Bonferroni correction for multiple comparisons was applied (*p* ≤ 0.006). All analyses were performed using SPSS v. 23.0 (SPSS, Inc. Chicago, IL, United States).

## Results

### Comparison of Behavioural Measures Among Age Groups

The Kruskal–Wallis *H*-test show: (i) a significant main effect of the age group on the task assessing the body schema (the Hand Laterality Task; H = 6.79, *p* = 0.033) and on the respective control task (the Object Laterality Task; *H* = 6.47, *p* = 0.039), this effect was not significant after applying the Bonferroni correction (*p* ≤ 0.006); (ii) a significant main effect of the age group on the task assessing the body structural representation (FBE; *H* = 59.92, *p* < 0.0001), but not on the respective control task (the Christmas Tree Task; *H* = 1.07, *p* = 0.586); this effect was significant also after applying the Bonferroni correction (*p* ≤ 0.006); (iii) a non-significant main effect of the age group on the task assessing body semantics (Object-Body Part Association Task; *H* = 1.30, *p* = 0.520), and on the respective control task (Object-Room Association Task; *H* = 2.03, *p* = 0.362).

The pairwise comparisons performed by means of the Mann–Whitney *U*-tests showed that scores obtained on the body schema and its control task, and on the body structural representation were significantly lower in the group of participants aged over 60 years than in the two groups of younger participants aged 18–40 years (Hand Laterality Task: *U* = 679, *p* = 0.022; Object Laterality Task: *U* = 702, *p* = 0.034; FBE: *U* = 64, *p* < 0.0001) and 41–60 years (Hand Laterality Task: *U* = 679, *p* = 0.023; Object Laterality Task: *U* = 692, *p* = 0.026; FBE: *U* = 119, *p* < 0.0001).

The pairwise comparisons for the FBE were significant also after applying the Bonferroni correction (*p* ≤ 0.006).

**FIGURE 2:**
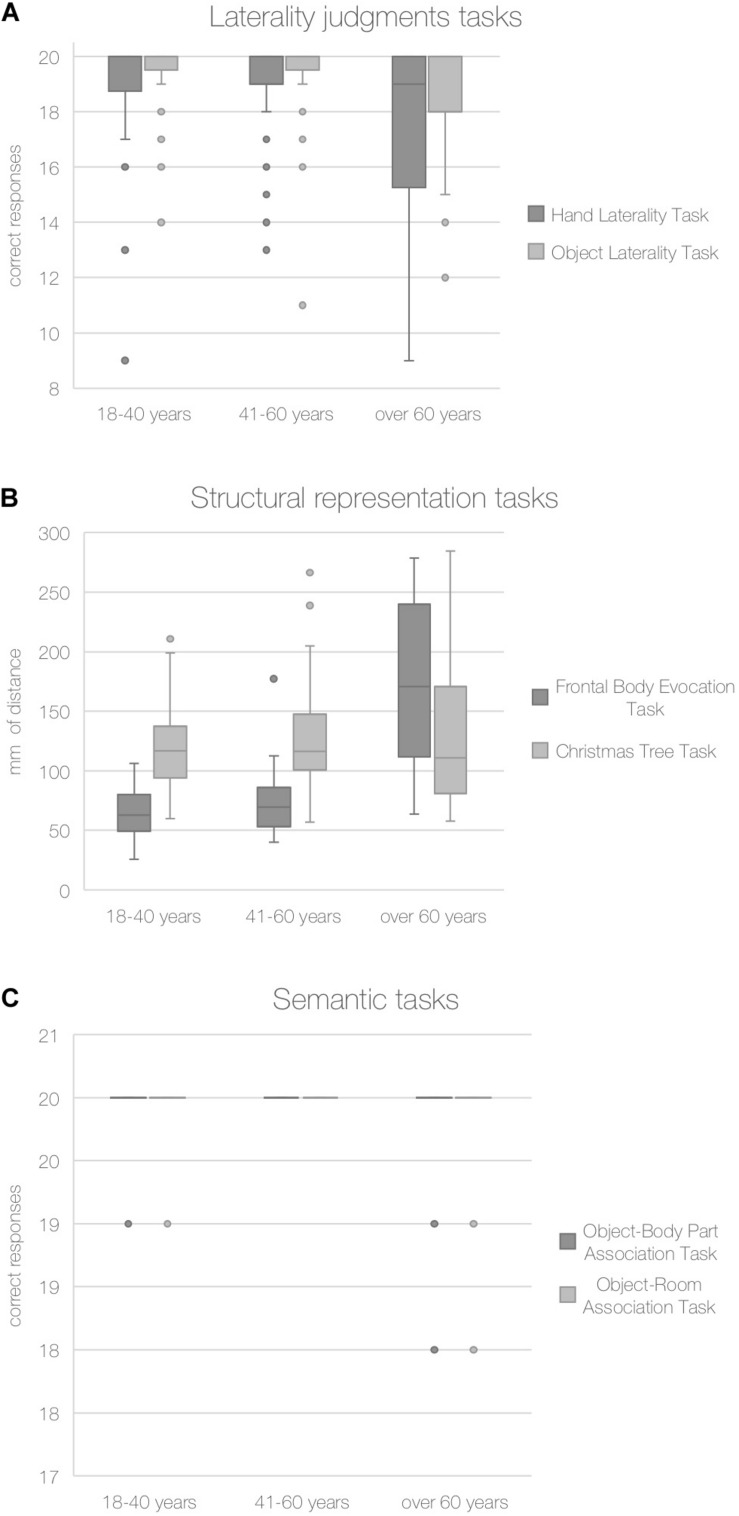
Accuracy in body and non-body tasks. Note: Median number of correct responses for laterality judgments **(A)** and semantic tasks **(C)** and mm of distance from the correct location for structural representation tasks **(B)** are reported for each age group.

The accuracy on the BR and control tasks for each age group are shown in Figure 2.

Given these results, to better investigate if aging had only an overall effect on the ability to mentally rotate stimuli (Object Laterality Task) or a specific effect on the ability to mentally rotate body parts (Hand Laterality Task), we performed a Wilcoxon test to compare performances on the Hand Laterality Task and Object Laterality Task of the older age group (over 60 years). Older adults showed significantly lower performances on the Hand Laterality Task (*Z* = −2.128, *p* = 0.031).

The analysis on the total SAQ scores showed no significant differences among the three age groups (χ2 = 2.39, *p* = 0.302).

**FIGURE 3:**
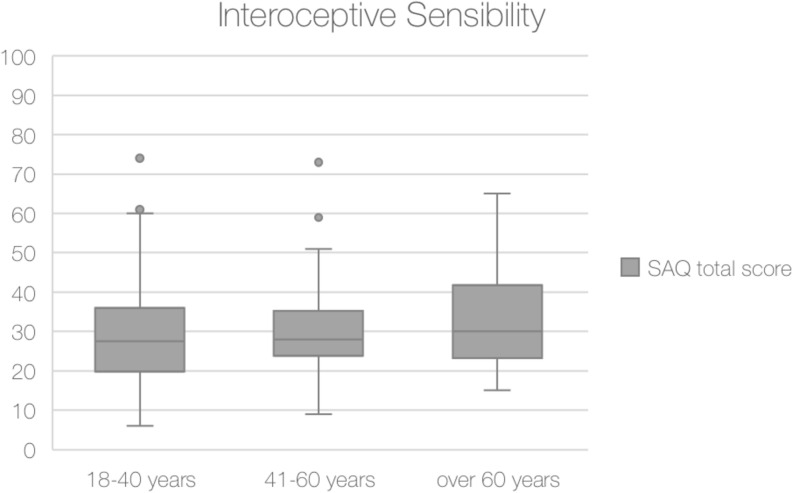
Median total score of Self-Awareness Questionnaire (SAQ) obtained for each age group.

Comparison of median total score of SAQ score among three age groups are shown in Figure 3.

### Correlation Between IS and BR Through the Adult Lifespan

The correlation analysis performed to assess the association between age and SAQ total scores showed no significant correlation. Similarly, no significant correlations were found between age and all control tasks (Object-Room Association Task, Christmas Tree Task, and Object Laterality Task), and between age and the body semantics task (Object-Body Part Association Task). Instead, significant correlations were found between age and the body schema (Hand Laterality Task) and between age and the body structural representation (FBE) task scores (for more details, see Table 2). In brief, we found that the older the age, the worse the participants performed on the tasks assessing the body structural representation and body schema (*p* ≤ 0.013).

**TABLE 2:** Correlation between age and SAQ total scores, BR and control tasks scores in the whole sample.

		Age	SAQ	Hand laterality task	Object laterality task	FBE	Christmas tree task	Object-Body part association task	Object-Room association task
Age	*r*_*rho*_		0.11	–0.23	–0.09	0.54	0.17	–0.01	0.01
	*p*		0.183	0.013*	0.267	0.001**	0.057	0.939	0.954
SAQ	*r*_*rho*_			–0.19	0.08	0.21	0.15	0.01	0.08
	*p*			0.024*	0.325	0.014*	0.070	0.844	0.307
Hand laterality task	*r*_*rho*_				0.39	–0.37	–0.29	0.18	–0.02
	*p*				< 0.0001**	< 0.0001**	0.001	0.030*	0.777
Object laterality task	*r*_*rho*_					–0.23	–0.13	0.11	0.08
	*p*					0.011*	0.141	0.205	0.315
FBE	*r*_*rho*_						0.32	–0.10	–0.05
	*p*						< 0.0001**	0.252	0.501
Christmas tree task	*r*_*rho*_							–0.08	0.15
	*p*							0.327	0.075
Object-Body part	*r*_*rho*_								0.18
association task	*p*								0.024
Object-Room	*r*_*rho*_								
association task	*p*								

SAQ, self-awareness questionnaire; FBE, frontal body evocation task.

*p < 0.05.

**p ≤ 0.001.

The correlations performed separately on the three age groups show that only in the older adult group there was a significant correlation between the SAQ and the body schema (Hand Laterality Task), and between the SAQ and body structural representation (FBE) (for more details, see Table 3).

**TABLE 3:** Correlations between SAQ total score and scores on the three BR tasks for each age group.

		Hand laterality task	FBE	Object-body part association task
**18–40 years**				
SAQ	*r*_*rho*_	0.11	0.02	0.17
	*p*	0.456	0.897	0.235
Hand laterality task	*r*_*rho*_		–0.25	0.19
	*p*		0.080	0.178
FBE	*r*_*rho*_			–0.09
	*p*			0.534
Object-body part	*r*_*rho*_			
association task	*p*			

**41–60 years**				
SAQ	*r*_*rho*_	–0.01	0.10	0.08
	*p*	0.924	0.487	0.601
Hand laterality task	*r*_*rho*_		–0.13	0.03
	*p*		0.362	0.834
FBE	*r*_*rho*_			–0.10
	*p*			0.477
Object-body part association task	*r*_*rho*_			
	*p*			

**Healthy participants group aged over 60**				
SAQ	*r*_*rho*_	−0.68**	0.67**	–0.27
	*p*	<0.0001	<0.0001	0.108
Hand laterality task	*r*_*rho*_		−0.72**	0.39*
	*p*		<0.0001	0.017
FBE	*r*_*rho*_			–0.28
	*p*			0.118
Object-body part association task	*r*_*rho*_			
	*p*			

SAQ, self-awareness questionnaire; FBE, frontal body evocation task.

*p < 0.05.

**p ≤ 0.001.

Furthermore the partial correlations conducted to examine the relation between IS and BR controlling for age (47.5 ± 14.85 years) on the whole sample showed no significant partial correlation (*r* = −0.144, *p* = 0.103) between the SAQ (30.55 ± 12.96) and the task assessing the body schema (Hand Laterality Task; 18.34 ± 3.21). However, the zero-order correlation showed a statistically significant negative correlation between the SAQ and the body schema task (Hand Laterality Task; *r* = −0.181, *p* = 0.040). This indicates that age had a significant influence in controlling for the relation between IS and body schema. As regards the body structural representation, there was still a statistically significant positive partial correlation (*r* = 0.274, *p* = 0.002) between the SAQ (30.55 ± 12.96) and the task assessing the body structural representation (FBE; 96.41 ± 66.01) when controlling for age (47.5 ± 14.85 years). The zero-order correlation showed a statistically significant and positive correlation between the SAQ and the body structural representation task (FBE; *r* = 0.310, *p* < 0.0001). This indicates that age had no significant influence in controlling for the relation between IS and body structural representation. As regards the body semantics, there was no significant partial correlation (*r* = −0.074, *p* = 0.404) between the SAQ (30.55 ± 12.96) and the task assessing the body semantics (Object-Body Part Association Task; 19.94 ± 0.27) while controlling for age (47.5 ± 14.85 years). The zero-order correlation showed no statistically significant correlation between the SAQ and the body semantics task (Object-Body Part Association Task; *r* = −0.073, *p* = 0.407).

Moreover, the partial correlation analyses performed to verify if the association between IS and the BR remained significant regardless of cognitive abilities required to perform tasks per se showed no significant partial correlation (*r* = −0.164, *p* = 0.064) between the SAQ and the unstandardized residuals of the Hand Laterality Task scores on the Object Laterality Task scores while controlling for age. In contrast, the zero-order correlation showed a statistically significant and negative correlation between the SAQ and the unstandardized residuals of the Hand Laterality Task scores on the Object Laterality Task scores (*r* = −0.193, *p* = 0.028). Overall, these results confirmed that IS was significantly correlated with the body schema when controlling for other cognitive skills and that age had a significant influence in this relation.

Regarding the body structural representation, there was a positive partial correlation (*r* = 0.250, *p* = 0.004) between the SAQ and the unstandardized residuals of the FBE scores on the Christmas Tree Task scores while controlling for age, showing that the association remained significant regardless of age. The zero-order correlation also showed a statistically significant and positive correlation between the SAQ and the unstandardized residuals of the FBE scores on the Christmas Tree Task scores (*r* = 0.291, *p* = 0.001). Overall, these results confirmed that IS was significantly correlated with the body structural representation when controlling for other cognitive skills and that age had no influence in controlling for this relation.

Regarding the body semantics, there was no significant partial correlation (*r* = −0.080, *p* = 0.369) between the SAQ and the unstandardized residuals of the Object-Body Part Association Task scores on the Object-Room Association Task scores while controlling for age. The zero-order correlation showed no statistically significant correlation between the SAQ and the unstandardized residuals of the Object-Body Part Association Task scores on the Object-Room Association Task scores (*r* = −0.079, *p* = 0.373).

### Moderation Role of IS in the Relation Between Aging and BR

To verify if the effect of age on the BR was conditioned by IS levels, a model 1 (i.e., a simple moderation model to test the effect of one independent variable on another dependent variable, conditioned on a third moderator variable, see Figure 1A) in the PROCESS macro for SPSS was run.

For the body schema (Hand Laterality Task), the overall model was significant (*R*^2^ = 0.13, *F*(3,133) = 2.971, *p* < 034) with a significant interaction between age and SAQ scores (*b* = −003, *t* = −2.35, *p* = 0.021). After controlling for cognitive functioning (Raven’s Colored Progressive Matrices scores), the overall model remained significant (*R*^2^ = 0.31, *F*(3,133) = 14.81, *p* < 0.001), as well as the interaction between the age and SAQ scores (*b* = −0.002, *t* = −2.10, *p* = 0.044). Then, these results showed a total direct effect of SAQ score on age and body schema (see Figure 1B).

For the body structural representation (FBE), the analysis indicated the overall model was significant (*R*^2^ = 3.70, *F*(3,133) = 22.91, *p* < 0.001); however, the interaction between age and SAQ scores was not significant (*b* = 0.037, *t* = 1.38, *p* = 0.169).

For the body semantics (the Object-Body Part Association Task), the analysis indicated the overall model was not significant (*R*^2^ = 0.02, *F*(3,133) = 0.674, *p* < 0.569).

**FIGURE 4:**
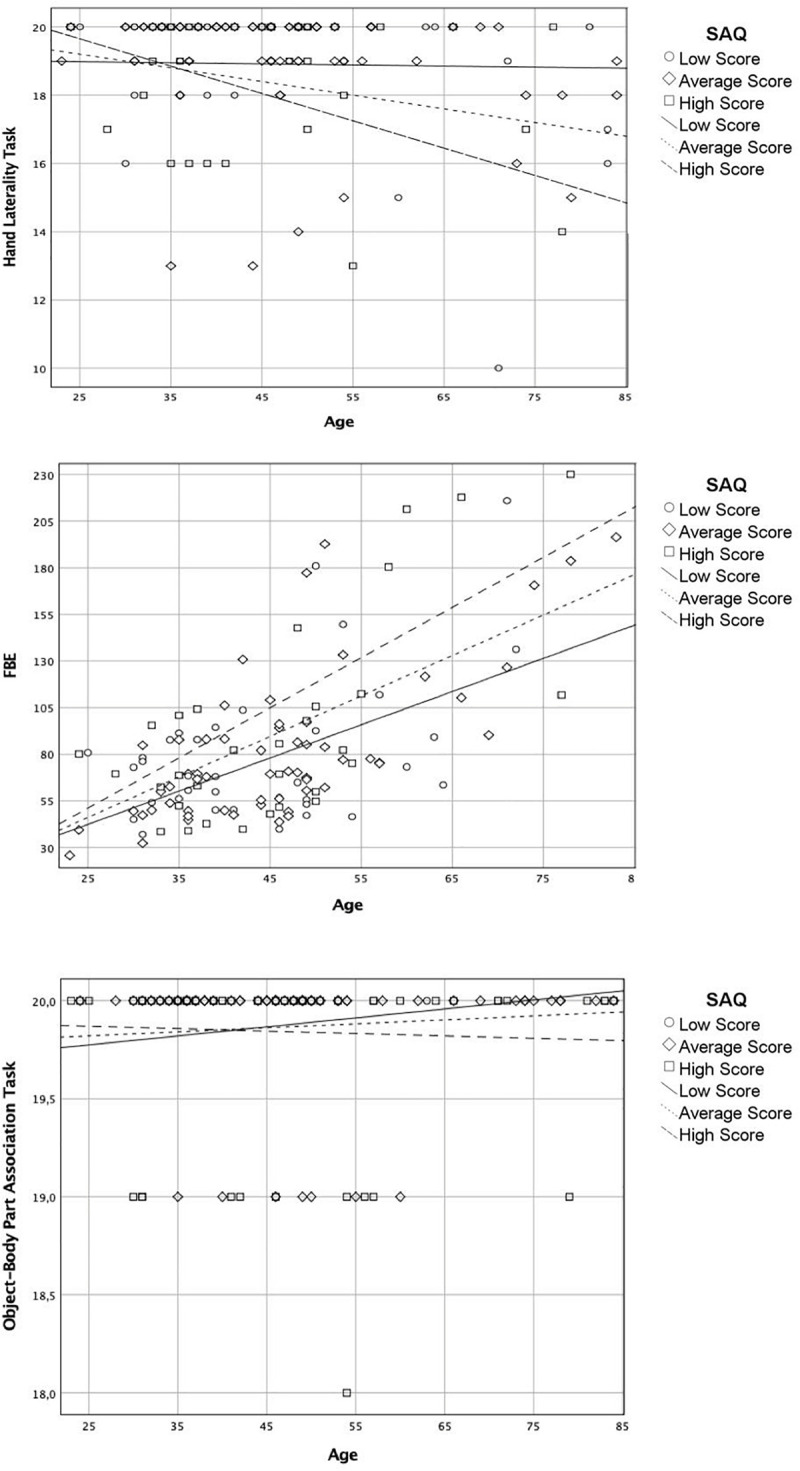
Moderating effect of the interoceptive sensibility on the association between age and body representation tasks.

Accordingly, IS appeared to moderate the relation between age and body schema significantly. The moderating effect of IS on the association between age and BR tasks is showed in Figure 4.

## Discussion

The present study aimed to investigate the relation between IS and BR during the adult lifespan. As a corollary, we also investigated the aging effect on IS and BR per se. The main finding of the present study is that higher levels of IS would worsen the performance on body schema with advancing age. Indeed, results revealed an age-dependent significant negative association between IS and body schema. In contrast, a significant association was found between IS and the body structural representation regardless of age, and no significant association was found between IS and body semantics.

At a closer look, IS also significantly moderated the relation between age and the on-line sensorimotor representation of the body (i.e., the body schema, see Figure 1B). Thus, greater difficulties to mentally rotate body parts would be affected by higher IS levels with increasing age, whereas the ability to localize and position correctly body parts (i.e., body structural representation) would be correlated to IS throughout all the adult lifespan and not moderated by it. So, this result would suggest the existence of a specific connection between IS and body schema with advancing age, rather than between IS and all the BR. In particular, the significant relation between IS and the body structural representation found in our correlation analysis could be mediated by the body schema, as these BR result related to each other (see Table 2). This possibility fits well with the theoretical co-construction model of BR (Pitron et al., 2018) that suggests that although the body schema and the body image are functionally distinct, their construction is partly based on their interactions and that the body schema, based on multisensory signals and motor expertise, influences the construction of body image (i.e., the body structural representation and the body semantics).

The specific connection between body schema and IS could be interpreted in light of previous research by Tsakiris et al. (2011) that in a sample of young adults (mean age 21.5 years) using the well-known RHI found a negative correlation between interoceptive accuracy (another component of interoceptive processing assessed by means of a heartbeat monitoring task) and body ownership over a fake hand, with low interoceptive accuracy resulting in a stronger sense of body ownership. As outlined in the introduction, body ownership also depends on the “coherence of current sensory input with pre-existing cognitive representations of the body” (Costantini and Haggard, 2007, p. 230); following Tsakiris et al. (2011) interoceptive accuracy may predict the malleability of the pre-existing BR. A higher awareness of one’s inner body sensations would decrease the plasticity of the BR and make it more difficult to feel ownership for artificial body parts that do not pertain to the physical configuration of the actual body. Further evidence of the body schema plasticity can be found in studies with tool use paradigms (Maravita and Iriki, 2004; Cardinali et al., 2009, 2012). Indeed, when actions are performed with tools, the morphology and functionality of specific body parts is modified through a quick and efficient updating of the body schema that allows the maintenance of action accuracy (Cardinali et al., 2009, 2016).

The association between IS and BR in aging could also be due to a reorganization of functional brain networks (Steffener et al., 2012; Bagarinao et al., 2019), and in particular to a decreased within-network and an increased between-network connectivity (Tomasi and Volkow, 2012; Betzel et al., 2014) in the visuospatial, the sensorimotor and the salience network (Bagarinao et al., 2019) associated with BR and interoceptive processing (Takeuchi et al., 2016; Chong et al., 2017). Indeed, studies using graph theory to identify the age-related changes in the brain’s network topology, found age- and aging-related decreases in the global measures of integration, segregation, and distinctiveness of the salience and sensorimotor networks (Chan et al., 2014; Shah et al., 2018).

Moreover, unlike previous studies (Khalsa et al., 2009; Murphy et al., 2018) that investigated interoception in older adults, we did not find a significant effect of age on IS likely due to a different method to assess interoception (i.e., a self-report questionnaire rather than a heartbeat detection task, see Khalsa et al., 2009) and to a difference in the age of the assessed samples (participants aged up to 84 years old compared with younger groups in the present study, rather than participants aged up to 90 years old as in Murphy et al., 2018).

Conversely, we found a significant age effect on BR tasks probing the body structural representation and body schema. These results expand on previous findings that point to an age effect on the body schema (e.g., Personnier et al., 2008; Skoura et al., 2008; Saimpont et al., 2009), on the experience of ownership toward an artificial and external limb, such as a rubber hand (Marotta et al., 2018), and on the ability to predict sensory consequences of one’s actions (Wolpe et al., 2016), highlighting how the multisensory integration and flexibility of BR may change across the lifespan (Marotta et al., 2018). Also, current findings are aligned with previous neuroimaging studies (McGinnis et al., 2011; Muller et al., 2016; Zhou et al., 2020) that found in older adults cortical thickness, decreased fractional anisotropy, and reduced functional connectivity in brain areas (i.e., insula cortex, primary sensorimotor area, inferior frontal gyrus, superior temporal gyrus, supramarginal gyrus) that are also damaged in patients with clinical conditions that affect BR (Baier and Karnath, 2008; Heydrich and Blanke, 2013; Boccia et al., 2020).

In conclusion, our results provide evidence regarding the relation between IS and BR during the adult lifespan. Nevertheless, some limitations should be acknowledged, such as the smaller sample size of the older group (37 participants over age 60 of which only six participants over age 80), and the sole use of the SAQ to assess IS, focusing mainly on negative visceral and somatic sensations (e.g., “I feel a burning sensation in my stomach;” “I feel that I can’t get enough air into my lungs”). Moreover, since almost all participants obtained the maximum possible score on the body semantics task (i.e., ceiling effect), results should be interpreted carefully, considering that the task used to tap body semantics may not be sensitive enough to age-related changes.

Thus, starting from our findings, further studies should enroll a larger sample size of older adults and consider and better investigate the possible moderator and mediator role of other psychological and physical variables (i.e., health anxiety and pain) and their clinical implications for the health maintenance in geriatric populations. Also, future lifespan studies should verify, in the same sample of participants, the effects of different components of interoceptive processing (i.e., interoceptive attention, interoceptive accuracy and IS) on different BR as well as on body ownership, to better understand how far each of them affects the malleability of different aspects of the cognitive body processing.

## Data Availability Statement

The raw data supporting the conclusions of this article will be made available by the authors, without undue reservation.

## Ethics Statement

The study involved human participants and was approved by Local ethics committees (Calabria Region Ethical Committee, Catanzaro, Italy, and Ethical Committee of the University of Campania ‘Vanvitelli’, Caserta, Italy). The participants provided their written informed consent to participate in this study.

## Author Contributions

All authors had substantially contributed to the conception and design of the work, to the interpretation of data and to draft and revise the work for important intellectual content. SR, MB, AD, and MC participated in data acquisition and statistical analysis.

## Conflict of Interest

The authors declare that the research was conducted in the absence of any commercial or financial relationships that could be construed as a potential conflict of interest.
